# On Monomania

**Published:** 1856-10-01

**Authors:** 


### THE JOURNAL
OF
### PSYCHOLOGICAL MEDICINE
AND
### MENTAL PATHOLOGY.

OCTOBER 1, 1856.

### Part First.

### Original Communications.

### Art. I.—ON MONOMANIA.

It is related that Pinel, on one occasion, took a lady to look over the arrangements of the Salpétrière. Having passed through several wards, the lady suddenly stopped, and asked where the insane people were, and if she might not be allowed to see them. By this she meant the violent insane; and after visiting them, she would doubtless leave the hospital with the full conviction that there were *two* perfectly distinct forms of insanity at least —an opinion which the most cursory glance at the inmates of any such establishment would tend to corroborate.

This man was yesterday perhaps a man of science, of genius, of extended philanthropy, piety, and benevolence. Look at him, and listen to him now,—his irregular and disorderly actions, his incoherent, perhaps blasphemous words, in which you may nevertheless trace the partial mechanical reproduction of vanishing ideas,—his indiscriminate, aimless violence—all betray a mind utterly overthrown, at war with itself and with the world. This woman was yesterday gentle, modest, affectionate, and fulfilled all the relations of life with prudence and propriety. See the contrast: "her timidity is changed into boldness, her gentleness to ferocity, her conversation consists of abuse, obscenity, and blasphemy; she respects neither the laws of decency nor of humanity; her nudity braves all spectators; and in her blind delirium she menaces her father, strikes her husband, or strangles her child."* A little while, and this will have passed away, but to

* Esquirol.

leave the unhappy victim a wreck, a caricature upon intelligent life. Pass on to another ward, and enter into conversation with that handsome, intelligent-looking grave gentleman in black. He evinces a mind well stored with ancient and modern lore; he can illustrate any subject upon which you enter with the riches of information and fancy; he discusses the current topics of the day—politics, art, or science, with considerable insight into their principles. Perhaps you detect no disorder of mind, until the close of an interesting interview, when, as a secret, he tells you of visions and special revelations from above, relating to some trivial matter of daily life; or he communicates, under strict injunctions not to betray him, his imperial rank, of which his enemies have deprived him; or his superhuman power, which he only refrains from using because of his love for his fellow-creatures. He is Socrates, and can give you most convincing arguments that he is entitled to be alive now; he is Jesus Christ, and is again subjected to the malice of the world; he is Mahomet; he is Napoleon Bonaparte, escaped forty years ago from St. Helena, and has been living here incognito ever since, for fear of re-banishment,—perhaps he retains his youth after all this, because he has discovered the elixir vitæ. Or he has squared the circle, and is imprisoned for envy by men of science; or he has the philosopher's stone, and is confined because of the political and social influence he might exert through its means.

Look again at that decent mechanic: a few weeks ago he was recognised as a hard-working, honest, upright member of society; but one day he seized a fellow-workman by the throat and strangled him. He had no quarrel with him, and liked him very much, he will tell you; but "he was not humble before God, and the blessed Trinity suddenly appeared and told him to kill him."* Yet he is mild and amiable in character, and talks with perfect coherence upon all matters within the scope of his intelligence. His neighbour evinces no morbid propensity, save that of setting buildings, &c. on fire. The next has made frequent attempts at suicide, the reasons for which are utterly trivial and futile. That wretched-looking creature in the next room laid violent hands on a favourite child, and then delivered herself up to justice. She did not know why, but the temptation to kill it was irresistible, and she yielded; with the completion of the act her right mind returned, and she became conscious of the crime; no intellectual aberration can be traced.† Here is another, with a look of excitement and importance, who will overwhelm you with a volume of words without connexion; half-formed ideas, causeless and evanescent emotions struggle

* "Correspondenzblatt," 1855. † Vid. Case of H. Cornier.

feebly for expression. He has forgotten the past, feels but imperfectly the present, and thinks not of the future. Lastly, crouching on the ground, or shuffling aimlessly along, is one whose mental development is below that of the beasts that perish. Some of his instincts alone remain, or perhaps these are gone, and nothing remains but vegetative life. Speak to him, and if you attract his attention, the only answer is a vacant stare, or an inarticulate chuckle or cry. Hunger, thirst, heat, or cold, are alike indifferent; the lichen or the sponge have almost as much vitality as he.

Many of these differences are obvious enough, and have been recognised from the earliest times in which mental diseases have been the subject of investigation. Two great forms of insanity have generally been admitted—one in which all the faculties were disordered, and one in which some remained virtually or apparently sound. The earliest classification was equivalent to a division into mania and melancholy;* by this latter term signifying a delirium or error, partial in its nature and extent. Pinel and Esquirol were the founders of the modern views as to the varieties of insanity. According to them, Idiocy and total Imbecility involve the original absence, or the utter loss of the intellect; Dementia signifies the loss of coherence in the thoughts, with general enfeeblement of the will, the sensibility, *and* the intelligence: Mania, complete perversion of these faculties, generally with exaltation in energy of function; whilst Monomania (here including both Lypomania and Chæromania) signifies a *partial lesion* of the intelligence, *or* the will, *or* the passions. Whilst there is but little difference of opinion on the existence of the first three forms of mental aberration, it is quite otherwise as to the last; and according to the diverse aspects in which the well-marked phenomena have been viewed, and also according to the different preconceived theories as to the absolute and essential constitution of the mind in relation to the unity or diversity of its faculties, widely different have been the opinions expressed upon the nature or existence of Monomania, especially that peculiar and important variety dependent upon lesion of the passions or instincts alone, and called *manie raisonnante*, moral insanity, and insanity without delirium. A dis-

* Wherever the word "melancholy" is made use of in ancient treatises or observations on insanity, and even in most modern ones, it must be observed that it differs entirely in signification from its social and conventional application. It was, and is yet frequently, used to signify a partial aberration of mind; in many instances, but by no means invariably, attended by depression of spirits. Its etymology is one of the very numerous relics of exploded theories, with which science in general, but ours especially, abounds. In fact, the terminology of mental science is rife with similar instances:—*melancholy* signifying black bile—*folly*, being derived from a word signifying a balloon full of wind—*lunacy and mania* both having reference to a lunar origin, &c. &c.

cussion on this subject, involving medico-legal principles of the very highest importance, has been carried on before the "Société Medico-Psychologique" during one entire year, in which the greatest authorities have taken part, in which the most diametrically opposed views have been promulgated, in which learning and candour have both been lavishly expended, yet in which, to use the words of M. Pinel (neveu) "*Chacun est resté avec ses convictions, sans que les arguments et les raisons de ceux qui pensent différemment aient pu le faire changer de manière de voir.*" The conclusion of this long discussion (of which an abstract is contained in the last number of this journal), and the publication of a monograph by the above-named M. Pinel, marked by great ability and research, suggest to us the opportunity of inquiring into this subject (on which we in England are not more united in opinion than our continental brethren, and in reference to which our practice is perhaps still more undecided), and of illustrating it by a few cases on which judgments have recently been pronounced on the continent.

The question hinges upon what is called the "solidarity," the absolute mutual dependence of the faculties of the mind, the oneness of the mind itself. The supporters of the theory of monomania speak of the mind as *compound*, consisting of many distinct faculties or attributes, any one of which may be disordered or subverted independently of the others. The opponents of this theory say that the mind is *one* absolutely, and if deranged, is deranged as a whole; that there may be a *prominent* morbid idea, but that this is only a symptom of a *universal* morbid state. Thus melancholia—which includes melancholia proper, or partial depressive delirium, and chæromania or monomania proper, partial expansive delirium—is described by Pinel as "delirium exclusively on one subject, no propensity to acts of violence, independent of such as may be impressed by a predominant and chimerical idea—*free exercise in other respects of all the faculties* of the understanding." Mania without delirium, "manie raisonnante," moral or instinctive insanity, which is essentially included in this question as hypothecating a derangement of *one order only* of faculties, the *affective*, is described by the same writer as involving "no sensible change in the functions of the understanding, but perversion of the active faculties, marked by abstract and sanguinary fury, with a blind propensity to acts of violence."* It is the existence of this form

* The opponents of this view either consider all acts of this nature as instances of ungoverned passion; or allowing the insanity, they consider it universal, violence being only the *predominant*, but not the *exclusive* morbid tendency. That many of the faculties of the mind are in what appears to be healthy action, is undeniable; but this fact is accounted for by saying that they are automatically performed.

of monomania which lends a great part of the practical interest to the question, on account of its medico-legal relations. M. Pinel (neveu) says :—"If it is of little consequence, *pathologically* speaking, whether true monomaniacs exist or not, it is otherwise when we examine the question in its relations to jurisprudence ; and it is under this point of view that the doctrine merits a serious examination." To illustrate the difference practically between the two contending parties, we suppose that a man commits a murder, and from the utter apparent absence of motive and other circumstances, the question arises as to his sanity. The opponents of the monomania theory examine him. After the most careful investigation, they can detect no present error *of the intellect*, nor any past evidence *save the crime itself ;* he is therefore pronounced sane and *criminal.* The supporters of the theory of monomania recognise as an element in their calculations the possibility of a blind impulsive fury arising in an otherwise sane mind, without clear* warning, and transitory in its nature, passing away with the completion of the act, leaving no trace. The absence of past or present intellectual disorder does not necessarily prove that he was sane at the time of the completion of the crime; and he is not pronounced criminal until after a careful inquiry into all his antecedents, his hereditary tendencies, and his general physiological condition. Esquirol, defining monomania as delirium on *one subject only*, recognises also most fully the disorder of the impulsive faculties without any lesion of the understanding:—"The impulse is sudden, instantaneous, unconsidered, *stronger than the will ;* the murder is committed without interest, without motive, most frequently upon those most dear to them."

Such are the opinions held now by one party. Of the other, some object to the word monomania, some to the existence of the thing itself, some to its legal applications; the first class denying that *one* morbid idea ever constitutes the whole affection, suggest the word "Oligomania" instead. The word is not of consequence ; but the opinions of the other classes are more important. A few quotations will indicate their view of the question.

M. Moreau says :—"According to the fundamental laws of the intellectual faculties, it is impossible to admit that these faculties can be modified in a partial manner. In the lightest, as in the most severe of these lesions, there is necessarily a *complete metamorphosis*, a radical and absolute transformation of *all the*

* There is often traceable, (as will be noticed afterwards,) *some* evidence of premonitory disorder, occasionally mental, but more frequently physical, as heat or *boiling* in the head, strange anomalous sensations in the stomach, or bowels, &c., but generally of so slight a kind as only to be remarked upon *after the fact.*

*mental powers* of the 'ME' which combines them."—"In other terms, as we reason, so we rave—a man is mad or *not mad;* he cannot be half or three-quarters insane, deranged in full face or profile." This opinion has the merit of being clear and distinct in its meaning, though, it must be confessed, rather startling.

M. Falret is one of the most uncompromising opponents of the doctrine of monomania. "He has even," says M. Pinel, "in full conclave of the Academy, defied any one to show him a single case." He says somewhat boldly that the believers in the doctrine "ne savent pas reconnaitre le fond maladif sur lequel se developpent et se perpetuent les idées prédominantes"—a conclusive statement, though scarcely an argument; and one admitting of no reply.

M. Foville does not deny absolutely the existence of monomania, but limits it extremely. In 1829 he writes thus:—

"Monomania consists in a delirium, partial and circumscribed to a small number of objects. Monomania in its most simple condition is excessively rare; the number of patients who only rave on one subject is *infinitely small* compared to the number of those who are *called* monomaniacs. Under this head are often confounded all those who have some habitual dominant idea. I have only seen two cases which rigorously merited the name; and these two even were affected from time to time with more extended delirium."

In 1834 he expresses himself even more strongly:—

"Let any one examine the hospitals of Paris, of Bicêtre, of Charenton, and he will see that amongst the thousands of insane there is scarcely one *true monomaniac—perhaps not one.* Insanity attacks principally at one time the intellectual, at another the moral or affective faculties, and again the sensations and movements. Each of these may be more or less profoundly affected than the others; and so when the intellect, *without being unaffected*, is less deeply involved than the other faculties, we fall into the error of considering it sound, and calling these monomaniacs. Indeed, it seems to me as though the descriptions of monomania had been written *upon the word*, and not from nature; that is to say, that writers have described what *might* merit the title of monomania, but of which they can find no instance in practice."

These are opinions on monomania in general; but when the question is concerning its criminal relations, the expressions are much stronger. M. Marc relates that on one occasion a magistrate said to him:—"If monomania be a disease, it ought, when it proceeds to capital crimes, to be cured in the 'Place de Grève'—that is, by the guillotine." Another opinion was the following:—"Monomania is a modern resource to screen criminals from the law, or to deprive a citizen of his liberty;—if he is not criminal, then he is mad, and must be sent to an asylum; or, if he be criminal, he may be preserved from punishment by the same plea."

Sir A. Morison says:—

"Had Oxford, Souchet, Briney, Ovenstone, and others been *punished*, whether by death or otherwise, I leave for others to determine, and cannot help thinking that we should not hear any more of 'irresistible impulse' in the production of crime. There can be no security for life, if the consequences of an act may be evaded by metaphysical conjectures on the strength of morbid impulses, and the impossibility of controlling evil passions. There is not a crime for which, with some show of reason, the excuse might not be made—'I did it because I could not help it.'"

Such are a few of the various opinions entertained as to the existence and nature of monomania. But the differences do not end here. Even among those who admit the affection, there is much diversity as to the amount of responsibility involved in any crime committed by persons so affected. Some consider that a well-ascertained insane idea absolves the subject of it from all *legal* responsibility; others, that the act must be within the province of the delusion; others, again, that even well-marked mania is not always a protection.* M. Delasiauve says that the incompatibility of moral liberty with a morbid state of mind is *not* absolute; and that they labour under a profound error who consider that all partial delirium implies necessarily the loss of free will, and base upon this circumstance their distinctions relative to legal responsibility. M. Gerdy rather amusingly attempts to cut the knot. He is—

"Astonished that men should introduce every moment *free will* into human action. Man is only perfectly *free* in indifferent matters! when his appetites, his interests, or his passions are in question, *liberty* is an empty word. What signifies the moral liberty of a robber who does not put his project in execution because he sees a policeman's hat? It is worth as much as that of the fox which does not attack the hen-roost because he sees the dog watching him."

As the subject of criminal responsibility will be fully treated in another place, we do not propose to discuss it here,† but

* See Dr. Damerow's opinions in the "foreign abstract" of last number.

† The following passages from M. Pinel's work claim attention, to render the subject more complete, and to give a fuller exposition of the diverse opinions entertained on so important a subject:—

"It is highly necessary to inquire, in those cases where the insanity assumes an intermittent form, if the individual possesses in the intervals entire command of the faculties, and enjoys *moral liberty;* if he is, in consequence, responsible. Indubitably, we cannot be too cautious in pronouncing upon such cases. Wherever well-defined alienation has preceded a criminal act, we ought to entertain at least the presumption of feebleness of free-will."—"De la Monomanie," p. 60.

"If we wish," says M. Pinel (the elder), "to interrogate the insane upon their condition, in general they elude our questions, or take refuge in obstinate reticence, inquire briefly into the *nature* of the affections comprehended under the names of Melancholia or Lypomania, Chæromania, or partial expansive delirium, monomania, mania without delirium, moral or impulsive insanity—all expressive of a disorder of some *one* faculty or order of faculties, whilst the others *practically* continue sound.

The differences of opinion on this subject seem to have arisen from the opposed methods of investigation made use of by the different observers. By some the strictly inductive method has been applied,—facts and phenomena have been carefully collated and analysed, and the results have been combined to construct a theory, or rather a formula; since the name given is rather a brief statement of the facts, than a theory to account for them. Hence, when a number of cases have been carefully analysed, in which the passions or appetites have been *morbidly* affected, whilst the intellectual faculties have been *apparently* quite unaffected ; or *vice versâ*, one of the intellectual manifestations has been observed to be uniformly in error, whilst the rest, and the volitional and moral faculties, have been *apparently* sound—

or they will answer perversely; it is only by studying their conversation and conduct for many months, by gaining their confidence, and inviting them to unburden themselves, that we can succeed in unveiling their most secret thoughts."—"Traité sur l'Aliénation," p. 58.

"It is truly distressing, and most astonishing, to see men, distinguished even for learning and prudence, attempt to support doctrines only founded upon erroneous theory, and opposed to the results of numerous and incontestable observations. It is, indeed, inconceivable that persons unacquainted with mental medicine or science, the study of which offers such difficulties, even to those who devote a life to it, will pronounce upon, and explain theoretically, and without ever having observed the insane, questions purely pathological."

"The alienists of all countries protest energetically against this illogical judgment of philosophers and lawyers upon a science of which they know not even the first elements."

"We have observed with pleasure that Dr. Forbes Winslow has entered a powerful protest against pretensions so unfounded, and especially against the opinion of certain English jurisconsults, who refused to recognise that partial insanity ought to involve irresponsibility. He writes with a just indignation:—

"'Partial insanity no valid excuse, no extenuation for crime! Partial insanity no plea, no justification in criminal cases! How monstrously unphilosophical, how wildly fallacious, how opposed to positive facts, how absurdly illogical, how grossly unjust, how repulsive, how abhorrent to every right-thinking, to every humane mind, and to every Christian and philanthropic heart! Apply this judicial, antiquated, and absurd dogma to the great mass of miserable and irresponsible lunatics at this moment legally in confinement, and two-thirds of them would be immediately made amenable to the law for their conduct! If partial insanity can be clearly established, who would be bold enough to declare or define the precise limits of the disease, or to sketch the boundary line separating a responsible from an irresponsible state of mind?'"—"Journal of Psychological Medicine," July, 1854.

"On the contrary, we have seen with pain that Dr. Mayo, a distinguished alienist of Great Britain, does not coincide in the opinion of almost all those who have devoted themselves to a study of mental pathology. The number for April, 1854, of the journal directed by Dr. Winslow, contains a very just criticism upon his doctrine."—"De la Monomanie," par M. Pinel, neveu, p. 61.

these have been called cases of monomania. On the other hand, the opponents of this theory have attempted the *à priori* system of investigation, and have reasoned from assumed cause to effect. They set out with the hypothesis, that the mind is *one and indivisible* (which may be very true); and that its faculties are absolutely mutually dependent (which seems to admit of doubt); and, therefore, that one faculty can *only* be perturbed by the whole mind being subjected to the disorder, (which appears to be contrary to observed facts.) So far it is only theory; but the converse practical application is more serious. The former party acknowledge that the instincts and impulses may be even criminally excited whilst the *mind* remains sound, and the subject be consequently considered irresponsible; whilst the latter, in the absence of any indication of a fundamental intellectual malady, consider these manifestations to be mere passion, and, therefore, criminal.

A few illustrations will serve to render the examination of these conflicting views more practicable.

For our purposes it may be sufficiently accurate to say, that the psychical manifestations of man develop themselves under the forms of understanding (or reason), volition, emotion, and instinct (including appetites, &c.). Each one of these may be the subject of *exclusive* disorder, according to some, of *predominant* disorder, according to others.

The understanding manifests itself in a variety of ways, (and this involves no theory,) as perception, conception, attention, memory, comparison, imagination, judgment, and others.

For purposes of illustration, we first select perception, which is universally recognised as a *mode of operation* of the mind. Now this class of phenomena require special organs for their manifestation, and we recognise the possibility of one or more of the divisions being entirely wanting, as the sight or hearing, without the mind being radically affected. But as that may be considered dependent only on physical conditions, we must inquire further into the general and universal perception. It is not necessary to do more than allude to those instances in which the senses are strong to the utmost point of refinement, or blunted almost to inertness; without the judgment being increased in the one case, or the imagination dimmed in the other—though these facts speak volumes against the "solidarity" of the mind, or the absolute mutual dependence of the faculties. But how does perception comport itself under *abnormal* circumstances?

The senses (on which perception is dependent) are subject to *illusions* and *hallucinations*. Selecting the latter for investigation, as less evidently dependent upon the *organs* themselves; we find—1. That they may exist independent of any mental derangement; 2. That they may be *one* symptom of such derangement, coexistent with many others; and 3. That they may *be*, or *constitute*, such derangement, the entire of the other mental phenomena being duly and healthily developed, so far as any known means of discovery serve.

Of the first position, two very striking instances are on record illustrating the persistence of all the reasoning faculties, whilst perception (or conception) was for the time utterly perverted. The first occurred in the person of Nicolai of Berlin, who relates himself how on one occasion, when suffering from mental agitation, he saw the figure of a deceased person standing in the room. He was naturally alarmed, and went into another apartment, where the figure followed him. By-and-bye, other figures appeared walking about, some of dead persons, but the greater number of the living; sometimes people of his acquaintance, but generally strangers. After some time they spoke, "either to one another, or to him., Their speeches were short, and never disagreeable." He saw them for two months, at home and abroad, in great numbers, even crowds; and whether his eyes were open or shut. He never from the first believed in the reality of these images. He could always distinguish the phantom from the reality, though the former had every semblance of the latter. "I knew extremely well," he says, "when the door only appeared to open, and a phantom entered, and when it really did open, and a real person came in." The calmness with which these *spectra* were observed, and the philosophic analysis of the laws of their appearance, and the ever distinct recognition of their unreal but *subjective* nature, are all very instructive, and indicate clearly that one faculty may be disordered, yet the others be lucid and acutely analytic.

Dr. Bostock refers to this case in his "Physiology," and recounts his own experience of something similar, when labouring under febrile symptoms and great debility, though, as he states, entirely "free from delirium."

"After having passed a sleepless night, I first perceived figures presenting themselves before me, which I immediately recognised as similar to those described by Nicolai; and upon which, as I was free from delirium, and as they were visible for three days and three nights, with little intermission, I was able to make any observations."

These spectra, unlike those of Nicolai, always followed the motions of the eyeball; and the most distinct of them were figures with which he had no acquaintance. Then followed a succession of images on a small scale, as it might be medallions, of objects or faces entirely unknown.

"During all this succession of scenery, I do not recollect that, in a single instance, I saw any object with which I had been previously acquainted; nor, as far as I am aware, were the representations of any of those objects, with which my mind was most occupied at other times, presented to me. They appeared to be new creations, or at least new combinations, of which I could not trace the original materials."

Not long ago a little boy, about six years of age, described to us, how he could not fall asleep at night, because he saw "lamps and sparks," and sometimes "faces looking in at the door." He was not alarmed. These were the consequences of a transient disorder, and completely passed away. The other senses are liable to be similarly affected. All these are instances of morbid perceptions, and perception is a mental faculty;—yet it would be an entirely gratuitous assumption, and one subversive of all useful generalisation, to hypothecate a fundamental derangement of the whole mind.

2. Hallucination, as one of many symptoms of insanity, is too frequent and well recognised to require much comment. The insane see visions, and hear voices, through which they live in a world quite apart from their fellows, and under the influence of which it is that most of their acts of violence are committed. A voice says "kill," and they obey blindly, impulsively. This form of hallucination most frequently attends the moral, instinctive, or impulsive alienation. Numbers of reports testify to the apparent soundness of the intellectual faculties, in cases where motiveless murders have been committed in obedience to a voice heard commanding the act. Doubtless, however, here, the intellect which acts upon this hallucination must be deranged in itself.

Some time ago, a gentleman was temporarily under our care, who had a strong tendency to mental disorder. At that time it was evidenced only by the idea, that all the company in which he happened to be at any time was laughing at him. Otherwise, the ideas were vigorous, and the thoughts deep and active. Very soon after, he became maniacal, and was confined for some months. As the extreme violence and delirium diminished, he obtained some knowledge of his situation, and actually simulated calmness so well, as to be set at liberty, under surveillance of a brother. His very first act was to contrive some means of temporary separation from his companion, and to blow his own brains out in the public road.

3. Hallucinations, *with a belief in their reality*, sometimes constitute the entire detectible derangement of the mental faculties. Nicolai and Bostock were sane—they knew perfectly well that their spectra were all *subjective*. M. Berbiguière, the author of "Les Farfadets," *the Goblins*, was insane: he had hallucinations, and believed in them; his imagination was so struck with the idea of goblins, that he saw them everywhere. He himself published an account of them in three large volumes. No mischief occurs but by means of these goblins—they interfere with his stable—they make his chimney smoke—they stop his watch—they make him sneeze. It is the goblins that cause bad weather—without them there would be neither hail, rain, nor thunder. We are deceived as to the causes of sudden death; we talk of apoplexy and the like; no such thing! it is the goblins that strangle them. The goblins put stumbling-blocks before people to trip them up—they are the cause also of many pregnancies in girls, whom others ignorantly suppose to have been seduced.

M. Berbiguière confesses that, sometimes these goblins occupy him so much that his *ideas become confused.* M. Baillarger has some pertinent comments on this case.

"If monomania must always be strictly limited to one false idea, assuredly Berbiguière was far from being a monomaniac. Yet the author of 'The Goblins' was assuredly neither a maniac nor an imbecile. There was no slowness nor prostration of faculties; on the contrary, he was an active and intelligent man, with *no incoherence* in his ideas. *Aussi a-t-il pu rediger un ouvrage de longue haleine, et le faire imprimer.*"

We have dwelt thus at length upon disorders of perception, as affording an illustration of the independence of the *elementary* faculties. Nor is this inquiry useless; for upon such hinges the entire question of the *solidarity* of the mind. If one faculty, or one class of intellectual faculties may be deranged, leaving the others intact; we cannot deny the possibility that those of passion or volition may in like manner be affected, the intellect remaining perfectly sound, its exercise overpowered or remaining in abeyance.

Disorders of subjective sensation afford additional argument for the comparative independence of the faculties. Cases of hypochondria are very frequent where the person is convinced that something living is in the body; yet he is fully competent to the discharge of all the duties of life. A very striking instance is given by Dr. Prichard, from Jacobi.

"A man, in other respects rational, of quiet and discreet habits, laboured under the impression that a person was concealed in his belly, with whom he held frequent conversations. He often perceived the absurdity of this idea, and grieved in acknowledging and reflecting that he was under the influence of so groundless a persuasion, but could never get rid of it. It was very curious to observe how, when he had but an instant before cried 'What nonsense! is it not intolerable to be so deluded?'—and while the tears that accompanied those exclamations were yet in his eyes, he again began to talk, apparently with entire conviction, about the whisperings of the person in his belly."

To the same class belong those more rare cases where a man fancies himself a bottle, a crown-piece, a barrel, or anything equally absurd, or fancies that his legs are made of glass or butter. Sensation is impaired, comparison and judgment are inefficient on this one subject to correct the impression; yet all this does not prevent the individual from acting in other respects like a perfectly rational being, and fulfilling every relation of life in the most exemplary manner.

So far, then, as regards the elementary faculties, the question seems tolerably clear, without entering even upon an important branch of evidence derived from the over- or under-development of the various faculties in reference one to the other; where perception or memory, for instance, may be preternaturally acute and tenacious, whilst comparison or judgment may be almost reduced to inactivity.*

When, leaving perceptions and sensations, the derangement has reference to the reception of a morbid *composite* dominant idea into the mind, the question becomes by so much the more complex, as there are different elements in this idea; and for the soundness of the remaining faculties, viz. those not involved in the erroneous conception, we have to trust to our own powers of investigation, or to those of others, rather than clear obvious

* Striking instances present themselves constantly of excessive or deficient development of single faculties, without the others being in any way affected. Frequently, also, certain parts or *conditions* of a faculty are hypertrophied, if we may use the expression, the rest remaining normal. Thus in memory, one person may have an almost preternatural memory for words, for numbers, for places, or for persons exclusively, the faculty being almost *nil* in regard to the others. We are acquainted with a gentleman who will play over an elaborate piece of music, which will from that time be fixed in his mind; yet if he hears a sentence of twenty words, his utmost efforts would probably be insufficient to prevent half as many blunders in its repetition. After certain fevers, also, some branches of knowledge pass entirely from the mind, either temporarily or permanently, yet leaving the basis of the intellectual powers perfectly intact. Many interesting illustrations of this, and allied facts, are given by Sir A. Morison, in his Lectures on Insanity.

Imagination is another faculty which affords strong illustration of the independence of the various manifestations of mind. How frequently is this developed most vividly, even morbidly; where judgment, combination, memory, and reason in general, are even below the average. How frequently is it entirely wanting, or apparently so, when the mind is sound and active in every other respect. But to multiply instances would be endless, as the point of biography in general depends upon the predominance of certain faculties over others.

But more closely connected with our present purpose is the inverse ratio so frequently observed to exist between the development of the intellectual and the moral nature. Of this, a most striking instance is related in the Memoirs of the Duc de Sully, concerning the celebrated Servin, in whom intellect appears to have been carried to the extreme of cultivation and refinement; whilst morally he was "treacherous, cruel, cowardly, deceitful, a liar, a cheat, a drunkard, and a glutton; a sharper, a blasphemer, an atheist; in a word, an illustration of all the vices that are contrary to nature, honour, religion, and society."

induction. We select for illustration two cases in which the ideas were very persistent, and the intellect in other respects *apparently* very clear.

M. Trélat, at the Bicêtre, had under his care a patient whose life was devoted to the discovery of perpetual motion. After vainly striving against this conception, M. Trélat thought that perhaps the great authority of M. Arago might have happy results upon the patient. Arago, after being assured that insanity was not contagious, accepted the commission to combat the morbid idea. He and M. Humboldt had an interview with the individual, who on being assured that his idea was a delusion, burst into tears, lamenting the loss of his error. Scarcely, however, had he left their presence, than turning to the physician he said, " C'est égal, M. Arago se trompe, et moi seul ai raison."

The next case is one of a singular nature, related by M. Esquirol. Mademoiselle F., the subject of the affection, was accustomed to visit frequently an aunt. One day on leaving the house she manifested some inquietude lest she should unwittingly have carried off in her reticule some *object of value* belonging to her aunt. This became henceforth her dominant idea—she could not touch anything, especially money, lest *something of value* should adhere to her. It was in vain to represent to her that she could not retain a piece of money without its being perceived. "That is true," she would say, " my uneasiness is absurd and ridiculous, but I cannot rid myself of it." If her hands touched anything, she acquired the habit of rubbing them assiduously, lest *anything of value* should adhere to them. If her dress rubbed against anything, it was carefully shaken and examined, lest *something of value* should be hid in the folds. Before sitting down, she examined carefully the seat—if it was loose, she shook it herself, lest *anything of value* might adhere to her dress from it. Sometimes her fears arose to such a pitch that she dare not touch anything whatever, and her attendant had to put her food into her mouth. Her toilette lasted from one and a half to three hours, so careful was she to examine every article of dress, to rub every part of the body, and to comb out almost every hair, lest anywhere should lurk *something of value*. The same minutiæ are observed in every action of the day. M. Esquirol observes:

"She does not rave, there is no incoherence—she knows her condition, recognises the absurdity of her apprehensions and her precautions—she laughs at them, sighs and weeps over them; not only does she make efforts to overcome them, but points out the means, often disagreeable ones, by which she thinks it probable her fears may be quieted. She is witty, intellectual, and in good health. It would be impossible at any time to detect the least disorder of the sensations, reason, or emotions of this interesting patient."

In such a case it would be entirely gratuitous to assert that there was a fundamental aberration of the whole mind, but that M. Esquirol was not sufficiently acute to detect it.

M. Pinel (neveu) relates the case of the Marquis X., who has been monomaniacal for thirty years. He seems to have but one erroneous idea, viz., that he is destined to the throne of England.

The same writer says:—

"A distinguished literary character, affected by a very circumscribed monomania, with hallucinations, has lived twenty years without his malady altering in the least; he believes that by the aid of magnetism his enemies are drying his brain. During many years' sojourn in my establishment, he had written a work, and his conversation was perfectly sane, so long as the subject was not magnetism, or some of his former friends."

Such are a few of the innumerable forms in which the intellect may be partially affected; but monomania, in the great preponderance of cases, affects the emotions, instincts, or impulses.

"Under the influence of an illusion, or an hallucination, of a sensation in the stomach, of a bitter taste, of a strong odour; at the sight of a white powder, of a book on the subject of poisons, the idea of murder or poisoning arises suddenly. The hearing of a sermon, where perdition and hell are painted in frightful colours, striking the imagination of the credulous and fearful, causes them to believe themselves eternally lost. The news of a revolution, or of a political persecution, the loss of a trial, a hazardous speculation, reverses of fortune, by exciting great perplexity, suggest the idea of spies, of arrests, of ruin, of misery: the unexpected death of some dear friend or relative, producing the most profound grief, suggests ideas of murder or suicide, which last for years before they are yielded to: imprudent words, or a light and inconsiderate conduct, provoke the idea of treason, of which the consequences may be fatal. The reading of romances, sentimental conversations, voluptuous images, the too frequent association of the sexes, by exalting the imagination, are the origin of fixed ideas, which terminate in erotomania. By reading medical works, the idea may be engendered of a fearful disease, slowly destroying life. This thought occupies the mind fully, and terminates in hypochondriacal monomania. The exclusive idea of revisiting the native country, is the prelude of nostalgic monomania."—(Pinel, "De la Monomanie.")

"The *irresistibility* of certain acts, their spontaneity, the impotency of the will, are incontestable facts. In some cases the patients present no appreciable alteration of the intelligence or the affections; they are driven by a blind instinct, by something indefinable, by an irresistible power."

M. Brierre de Boismont, the last-quoted writer, seems *now* to hold opinions practically at variance with these, as he is a firm believer in the *solidarity* of the faculties, and even believes that those men who have any one passion strongly excited, must necessarily feel the physical, moral, and intellectual nature thereby disordered. It is somewhat singular that two of his illustrations of this position should be two men, who assuredly had at least *some* faculties in the highest and clearest state of activity—Rembrandt and Molière. There are many forms in which partial, moral, or impulsive alienation of mind manifests itself. We must content ourselves with indicating briefly the types, for the present referring our readers for illustrations to systematic works on the subject.

1. *Theomania,* or religious delusion. The subject conceives himself to be in a special manner the object of divine favour, or wrath. Religious ecstasy or terror take complete possession of him : he has visions of angels or of the Trinity ; he hears voices which command him to offer a human sacrifice, and he kills those who are nearest and dearest to him. Or it assumes a purely melancholy aspect, under the idea of final perdition. This is a very obstinate form of the affection.

2. *Demonomania.*—Two affections have been noticed under this name—one a true mania, the subjects of which were called Demoniacs; the other a monomania, in which the patients had the idea that they were possessed with a devil, and would use the most grotesque means to expel him; in other respects being apparently sane.

3. *Pantophobia.*—The subject of this form has the free exercise of reason in some respects, but is in a state of perpetual terror. He will take no food, lest it should be poison : every one whom he meets is an enemy watching him; every natural occurrence is an omen, &c. &c.

4. *Hypochondriacal Monomania.*—The varieties of the form are innumerable, all having reference to the physical or functional condition of the body. The sufferer labours under a fearful, an incurable affection; he has living creatures in his stomach, heart, or head; every sensation is connected with danger or approaching dissolution; or he is dead, or his sex changed, or he is some other animal. This last delusion has been classed by itself as Zoanthropia. Some interesting instances are recorded in Burton's "Anatomy of Melancholy."

5. *Erotomania,* or Love Madness.—This is a *sentimental* affection, as distinguished from the *physical* disorders known as Satyriasis and Nymphomania. It appears to have but little connexion essentially with sexual sensations. The mind is perpetually fixed upon one object; in its pursuit everything else is disregarded ; blows, ill-treatment, and neglect are all inefficient to cure the propensity. It gives rise to fearful jealousy, which may cause homicide. It most frequently terminates in suicide or general paralysis, and, next to Theomania, is the most obstinate of these affections. A very interesting case is related in the "Correspondenz-blatt" for February 29, 1856.

6. *Dipsomania.*—A maniacal insuperable tendency to the abuse of alcoholic liquors, very frequently accompanied by intense horror of the practice, but inability to conquer it. It is to be distinguished from mere drunkenness. M. Esquirol observes: —"Si l'abus de liqueurs alcooliques est un effet de l'abrutissement de l'esprit, des vices de l'éducation, des mauvais exemples, il y a quelquefois un *entrainement maladif* qui porte certains individus à abuser des boissons fermentées. Il est des cas dans lesquels l'ivresse est l'effet du trouble accidentel de la sensibilité physique et morale, qui ne laisse plus à l'homme sa liberté d'action." A medico-legal inquiry into a case of this nature, when the intellectual faculties were perfectly sound, will be found quoted from Dr. Eulenberg, of Coblentz, in the "Annales Medico-psychologiques" for January, 1856. It is an extremely obstinate affection.

7. *Pyromania.*—A morbid disposition of mind, leading to acts of incendiarism without any motive. This may be either impulsive or reasoning. It occurs chiefly, but not exclusively, in young people. M. Marc gives many instructive cases, and Dr. Fritsch, of Rössel, in Prussia, relates a case which became the subject of medico-legal investigation, in the "Correspondenz-blatt" for September, 1855, in which he gave the opinion that the boy was not mentally deranged, and consequently responsible. The question has frequently been brought before our courts of justice of late years, with varying results. This form is not so difficult of cure as some others.

8. *Kleptomania.*—A propensity to steal in an absurd and motiveless manner. A gentleman confessed to his spiritual director that in spite of all efforts he could not resist the tendency to appropriate what was not his own. The priest having permitted him to steal, under the condition that he *always returned the articles*, he was quite content, picked the priest's pocket of his watch during confession, and returned it immediately afterwards. Some most interesting cases clearly connected with morbid excitement are related by M. Renaudin in the "Annales Med.-psy." for April, 1855.

9. *Suicidal Monomania.*—Many forms of mental derangement are accompanied by a tendency to suicide: in some this morbid propensity seems to exist almost without any other delirium. This is also sometimes impulsive, sometimes reasoning, but very frequently imitative.* It often accompanies the homicidal variety. The curability of this affection, when well established, is always subject to doubt; as, from various well-authenticated cases, there seems to be no limit to the period of time which may elapse in apparent soundness; and yet the propensity may return, and the act be finally accomplished. It occurs most frequently in adults, but sometimes in children of the tenderest years, illustrations of which may be found in our "Foreign Abstract" for April. Interesting details and cases are also contained in Dr. Winslow's work on the "Anatomy of Suicide."

10. *Homicidal Monomania,* manie sans délire, manie raisonnante, fureur maniaque, moral and impulsive insanity.—Such are some of the names given to a most important affection, under the influence of which a person, in whose intellect no flaw or imperfection can be discovered, may be suddenly urged on to commit murder unpremeditated and motiveless, and be considered irresponsible according to law, because of unsoundness of mind. Here it is that the two parties above alluded to are most completely at issue; and it is probably from unwillingness to admit this form of mania, that the *independence* of the faculties of the mind (from which the existence of such a form is logically deducible) has been so strenuously denied, and the theory of "solidarity" invented. It must be confessed that both these views have difficulties of no ordinary weight attached to their reception; for whilst the adhesion to the one would have a tendency, if not applied with extreme caution, to shield criminals from punishment adequate to the crime, the adoption of the other would frequently lead to the execution of irresponsible beings. No question in the range of science can be of more importance than this—none more difficult—none requiring in each individual case more caution, knowledge, and acuteness in its determination. No wonder that some are disposed to cut so gordian a knot by the assertion that such cases are impossible.

M. Esquirol seemed in 1818 to believe that in *all cases* of homicidal mania there was some detectible trouble of the intelligence. In 1838 he corrects the opinion as follows:—

"Since that period (1818) *I have seen* mania without delirium, and I must submit to the authority of facts. The following observations demonstrate that if the insane, deceived by delirium, by hallucinations, or by illusions, kill; that if the insane, affected by *reasoning madness,* kill, after having premeditated and reasoned upon the homicide they were about to commit: there are others, monomaniacs, who kill by a blind instinctive impulse—they act without consciousness (*sans conscience*), without passion, without delirium, without motive;

* Vid. Art. on "Mor. and Crim. Epidemics."—April.

—they kill by a blind, instantaneous impulsion, independent of their will."

There are many varieties of manifestation of this tendency: some are influenced by chimerical and irrational motives; some have no motive at all, but feel impelled to commit the act of violence, against which they continually, and sometimes effectually, struggle. Conscious of their excited condition, they beg to be confined, or they implore the object of their blind fury to leave them. Some again, act suddenly, instantaneously; the will being overpowered by the impulse. There is no interest, no motive; most frequently the person killed is a child, or one most loved.

M. Esquirol concludes, 1. "That there is a homicidal monomania, sometimes accompanied by intellectual, sometimes by emotional disorder; sometimes by impotency of the will, which deprives the man of moral liberty."

2. "That there are signs which characterise this species of madness, and which, at least in the majority of cases, serve to distinguish insanity from criminality." The circumstances to be considered in forming a judgment on such cases are those preceding, during, and after the commission of the act. The constitution must be taken into account as to excitability; the habits of life as to peculiarity or eccentricity—something of this will often be found. The hereditary relations must be inquired into for traces of insanity. Those subject to this affection are generally found to have been of mild and amiable dispositions, and of religious impressions; but for some time, slight changes will have been remarked. The act may be coincident with puberty, or some physical irritation. Often preceding it, there may be traced headachs, and undefined disorders of sensation in the bowels and other parts. The crime may again be distinctly traceable to imitation, as before remarked.

At the time of the act, the differences between the criminal and the maniac are very strongly marked. The former kills from motive or interest, those who oppose his schemes, or whom he dislikes; the latter kills, without motive, those who are dearest to him. After the deed, the criminal flies, conceals himself, denies the fact, professes insanity sometimes, and adopts every conceivable method to screen himself. Nothing can be more opposite than the conduct of the monomaniac—he completes his murder, and the orgasm has passed away; he remains by his victim, and gives full details of the murder, aimless and motiveless.

Often the consciousness of the enormity of the act comes upon him with such force, that he attempts suicide, or delivers himself up to the hands of justice, imploring that he may be put to death, to escape from his despair. A careful and conscientious examination of circumstances like these would generally conduct to a correct judgment. The question at issue between the opposed parties on the subject of monomania, is as to whether these cases are instances of true derangement, or (in the absence of any detectible intellectual disorder) of mere unbridled passion and *unresisted,* but not *irresistible* impulse; and hinges upon the *independence,* or the "*solidarity,*" of the faculties of the mind.

We have found strong reasons for believing that the *elementary* faculties, at least, are in great measure independent of each other, in their *normal* condition. That A may be developed to the highest point, whilst B and C are almost non-existent; and that any one may be excited by casual circumstances whilst the rest remain unaffected. And as a considerable proportion of pathological changes are only permanent and exaggerated conditions of physiological states, it is reasonable to suppose that one of these faculties may be even morbidly modified, all the others remaining sound.

Observation and analogy alike inform us that the same holds good in the more complex operations of the mind, such as those involved in the ideas of relation, judgment, &c. Whether it is the same with regard to the *active faculties,* must be decided in some measure by observation, although analogy seems very explicit on the point. Now the most careful observers say that these are as independent as the former; and it must be remembered that our knowledge of mental phenomena is derived exclusively from observation. We have no *à priori* information on these subjects; the independence, and the solidarity of the faculties are alike theoretic, if not supported by observation. If, therefore, it is shown on good authority that one class of faculties, for instance the moral, are disordered, whilst the intellectual faculties remain sound, and that crimes are committed under morbid excitement of the impulses or instincts, which overpower and annihilate volition, whilst at other times no psychical aberration can be detected, we are compelled to believe in the independence of the faculties, unless we discredit the evidence. It is certainly possible to object, as does M. Falret, that there *is* a fundamental morbid change in the entire mind, but that the observers have not been acute or careful enough to detect it. To this there is no reply, as it would be a gratuitous assumption, and one of a nature to subvert all scientific evidence.

In conclusion we feel justified in believing:—

1. That the mental faculties are *virtually* independent, though very closely connected, functionally, in their normal condition.

2. That they may be morbidly affected, partially, or separately; one faculty being exaggerated, weakened, or perverted, whilst the others remain sound.

3. That such partial affection may constitute a disease properly enough termed monomania, or Oligomania, which may affect exclusively the intellect, the will, the appetites, or the passions, and that such cases are frequently met with.

4. That this disease is not a *period* of the more extended manias; but inasmuch as the connexion between the faculties is so close and constant, when one or a few have been disordered for some time, the delirium frequently, though by no means invariably, becomes extended, and the mania becomes general.

5. That crimes are committed of a serious nature under these conditions, for which the individual ought to be considered totally irresponsible; he being moved by the *same* unconscious and ungovernable impulse that many animals manifest under the stimulus of certain colours, odours, &c.

6. That motiveless, aimless, unnatural and anomalous crimes should always be carefully examined, as probably belonging to this class.

7. That much precaution is necessary in acknowledging the cure of any monomaniac once guilty of incendiarism, attempts at suicide, or homicide—though it is possible that such cure may in certain cases be complete.

The social relations of monomania, together with remarks on the diagnosis, prognosis and treatment, we defer for the present, our limits compelling us to pause.*

* The Editor of the "Psychological Journal" does not hold himself responsible for all the opinions expressed in this article.

